# Magnetic Resonance Imaging Features in Different Types of Invasive Breast Cancer: A Systematic Review of the Literature

**DOI:** 10.7759/cureus.13854

**Published:** 2021-03-12

**Authors:** Amer Alaref, Abdallah Hassan, Rajan Sharma Kandel, Rohi Mishra, Jeevan Gautam, Nusrat Jahan

**Affiliations:** 1 Diagnostic Radiology, California Institute of Behavioral Neurosciences & Psychology, Fairfield, USA; 2 Diagnostic Radiology, Thunder Bay Regional Health Sciences Centre, Thunder Bay, CAN; 3 Diagnostic Imaging, Northern Ontario School of Medicine, Sudbury, CAN; 4 Internal Medicine, California Institute of Behavioral Neurosciences & Psychology, Fairfield, USA; 5 Cardiology, Rush University Medical Center, Chicago, USA

**Keywords:** mammogram, breast cancer, idc, dcis, ilc, mri breast, ultrasound

## Abstract

Breast cancer is the most common malignancy affecting women worldwide, and early diagnosis of breast cancer is the key to its successful and effective treatment. Traditional imaging techniques such as mammography and ultrasound are used to detect and configure breast abnormalities; unfortunately, these modalities have low sensitivity and specificity, particularly in young patients with dense breast tissue, breast implants, or post-surgical scar/architecture distortions. Therefore, breast magnetic resonance imaging (MRI) has been superior in the characterization and detection of breast cancer, especially that with invasive features. This review article explores the importance of breast MRI in the early detection of invasive breast cancer versus traditional tools, including mammography and ultrasound, while also analyzing the use of MRI as a screening tool for high-risk women. We will also discuss the different MRI features for invasive ductal carcinoma and lobular carcinoma and the role of breast MRI in the detection of ductal carcinoma in situ with a focus on the utilization of new techniques, including MR spectroscopy and diffusion-weighted imaging.

## Introduction and background

Breast cancer is the most common malignancy in women worldwide, with a lifetime risk of 12.4%; an early breast cancer diagnosis is always the key to successful and effective treatment. Breast cancer detection at an early stage has always been a challenge, as small cancers are usually difficult to discover compared to larger ones, especially in young women who have denser breasts. The best modality to detect breast cancers is magnetic resonance imaging (MRI). Breast MRI has the highest sensitivity for breast cancer detection, ranging from 94% to 100%, and its ability to depict small invasive cancers measuring up to 5 mm [1]. Breast cancer accounts for nearly one‐third of all female cancer diagnoses in the United States and is responsible for approximately 20% of female cancer-related deaths [2,3].

Using breast MRI has been rapidly increasing as a screening tool for high-risk populations and as a problem-solving tool in indeterminate cases. It has been confirmed that breast MRI should be done routinely with intravenous (IV) gadolinium-based contrast agent injection. The only exception to IV gadolinium injection is when MRI is used to evaluate silicone breast implants for rupture or leak (intra-capsular and extra-capsular). Invasive breast cancer, especially lobular cancer, has always been diagnostically challenging, as it has an infiltrative pattern [3,4].

Invasive lobular carcinoma (ILC) and invasive ductal carcinoma (IDC) are the most frequent breast cancer subtypes. The most common subtype is IDC, followed by ILC. ILC represents a subgroup of 5% to 15% of all invasive breast cancers, with an increasing incidence during the past decade. Depending on conventional mammography and breast ultrasound, diagnosis of ILC is often challenging due to its diffuse nature appearing similar to normal breast parenchyma in radiographic mammography or ultrasound [5].

Non-mass enhancement (NME) on breast MRI is a term used for the areas that show enhancement without a mass in the pre-contrast sequence. Some of these areas are identified as malignant, including IDC, ductal carcinoma in situ (DCIS) and tubular carcinoma. Others are diagnosed as benign masses, such as sclerosing adenosis, atypical ductal hyperplasia, fibroadenoma and mastitis [6].

Traditional imaging techniques such as mammography and ultrasound are used to detect and configure breast abnormalities; unfortunately, these modalities have a low sensitivity and specificity particularly in young patients with dense breast tissue, breast implants or post-surgical scars/architecture distortions. Therefore, breast MRI has been superior in the characterization and detection of breast cancer, especially that with invasive features [7]. Moreover, women on hormone replacement therapy have a higher risk of breast cancer; therefore, MRI screening is crucial for discovering cancer [8].

This review article aims to explore the importance of breast MRI in the early detection of invasive breast cancers versus traditional tools, including mammography and ultrasound, while also analyzing the importance of MRI as a screening tool for high-risk women. We will also discuss the different MRI features for IDC and ILC and the role of breast MRI in the detection of DCIS with a focus on the utilization of new techniques, including MR spectroscopy and diffusion-weighted imaging (DWI).

## Review

Protocol

We followed the Preferred Reporting Items for Systematic Review and Meta-Analysis (PRISMA). The protocol was prepared but no registration was done as it was optional.

Inclusion/exclusion criteria

We decided to include the studies with the following inclusion criteria: free full-text papers, articles published within the past 10 years, human studies only, studies with the female population, articles published in the English language, studies including patients 19-44 years of age, and those aged 65 years and above. Animals studies and articles published in any other language were excluded.

Databases and search strategy

A comprehensive search was conducted using PubMed, Medline, PubMed Central, and Google Scholar, with the use of regular keywords and Medical Subject Headings (MeSH) keywords. The search was started on May 1, 2020, and continued until January 17, 2021. The following search strategy was used on PubMed including MeSH and field search: Breast Neoplasm OR breast cancer OR breast carcinoma OR breast tumor OR mamillary tumor AND imagining OR radiology AND (“Breast-Neoplasms/diagnosis”[Mesh] OR “Breast Neoplasms/radiotherapy”[Mesh]) AND (“Breast -Neoplasms/diagnosis”[Mesh: NoExp] OR “Breast Neoplasms/radiotherapy”[Mesh: NoExp]).

Study selection and screening

Two authors screened the articles after removing the duplicates and reviewed the articles by screening abstracts and titles. Any disagreement was resolved by discussion. Then the full texts of the articles were screened.

Data collection

The next step was data extraction; two authors independently extracted data using a data extraction form. Later the synthesis of data was done, and the writing process began.

Critical appraisal

Quality appraisal was done using the Cochrane Risk Bias Assessment Tool, Newcastle-Ottawa Scale for observational studies, and AMSTAR (A MeaSurement Tool to Assess systematic Reviews) scale, as necessary. Studies with a score of less than 70% were removed and studies with less bias were selected.

Results

**Figure 1:**
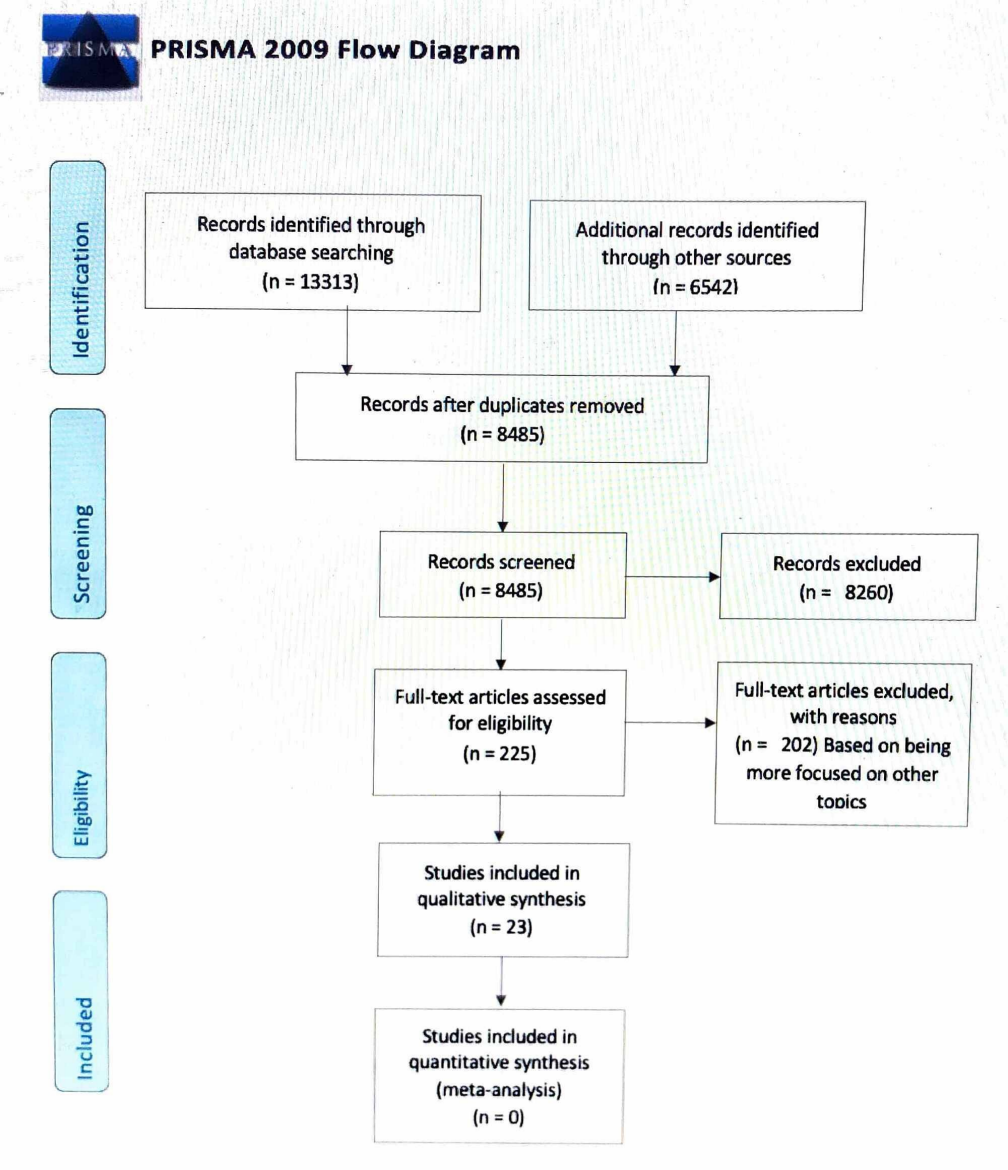
Selection process and the PRISMA flow diagram PRISMA, Preferred Reporting Items for Systematic Review and Meta-Analysis

The search yielded 13,313 records on PubMed. Combining all databases and grey literature, we found 19,765 papers. We removed the duplicates using Microsoft Excel. After duplicates were removed, 8485 papers were identified. After screening by reading the abstracts and titles, only those deemed relevant were kept; this resulted in 225 articles. We searched the full-text articles of the remaining 225 studies and removed those articles that did not meet our inclusion-exclusion criteria. Two authors did this process of screening. The exclusion was done based on different study fields, different situations, non-relevance, and different study designs, for example, those that did not assess for breast cancer and diagnosis through imaging or only focusing on ultrasound and mammography. Finally, we were left with 23 articles in PubMed for our review. The selection process is detailed in Figure 1 [9].

Discussion

On analyzing 23 published articles, we found that breast MRI is widely used to detect and characterize breast lesions incidentally discovered on routine screening (including mammogram and ultrasound) of symptomatic patients with palpable breast lesions. Also, surgeons have widely used breast MRI for the presurgical evaluation of already known breast cancers, including evaluation of invasion of the chest wall, nipple-areolar complex or skin, and multifocal or multi-centric cancer. The most critical clinical output of breast cancer's early discovery is to prevent distal metastasis when surgically removed. Pilewskie and King discussed the MRI features for breast cancer and the impact of MRI on short‐term surgical outcomes and local recurrence rates. In addition, they addressed the use of MRI in specific patient populations, such as those with DCIS, ILC and occult primary breast cancer, and discussed the potential role of MRI for assessing the response to neoadjuvant chemotherapy as well as the future perspectives of using MRI including higher field strength 7.0 T magnets [2].

Breast MRI as a Screening Tool

Breast MRI has been used as a screening tool in high-risk populations, as many women with specific genes and mutations are more likely to develop breast cancer. The European Society of Breast Cancer Specialists (EUSOMA) mentions that approximately 3% of all breast cancers occur in women with BRCA1 and BRCA2 deleterious mutations. A further small percentage occurs in women with TP53 mutations (Li-Fraumeni syndrome) or rare moderate-penetrance alleles such as CHEK2, ATM, and BRIP1, or low-penetrance more common alleles. BRCA mutation carriers and their untested first-degree relatives should be considered at high risk of breast cancer, with a lifetime risk of over 50%-60%. Moreover, they recommended annual MRI screening based on expert consensus opinion for women affected with Li-Fraumeni, Cowden, and Bannayan-Riley-Ruvalcaba syndromes, and first-degree relatives and those who underwent mantle radiotherapy who were under 30 years of age. However, the evidence is insufficient to recommend for or against MRI screening based on a 15%-20% lifetime risk for breast cancer and in women with lobular intraepithelial neoplasia, atypical ductal hyperplasia, heterogeneously or extremely dense breast on X-ray mammography (XRM), and personal history of breast cancer, including DCIS. Finally, they recommended against MRI based on the expert's consensus opinion for women at less than 15% of lifetime risk [3].

The Value of MRI in the Assessment of Invasive Lobular Carcinoma Versus Invasive Ductal Carcinoma

Mann studied the MRI features in ILC and compared them to IDC, concluding that even with the relatively small differences between IDC and ILC, diagnosis of ILC is really difficult and challenging because of the diffuse growth nature of ILC, compared to IDC [4]. ILC is larger than IDC due to the infiltrative process. ILC's diffuse growth nature causes conventional imaging (including mammography and ultrasound) to be non-dependable, as a result, causing a high percentage of tumor re-excision, forcing the surgeons and patients to proceed for mastectomy. Luckily, studies have revealed that mastectomy rates for ILC are reducing. Due to the breast MRI value in cancer staging, it plays a significant role in the preoperative staging of ILC patients. The genetic cornerstone for these dissimilarities is probably related to a mutation in the E-cadherin gene (CDH1). E-cadherin is directly linked to cell-cell unity and influences the morphology and motility of the cells. A lack of E-cadherin declaration may be responsible for the disconnected growth of ILC. Aside from the lack of E-cadherin declaration, classic ILC mimics low-grade IDC biologically. Similarly, the more aggressive type pleomorphic ILC mimics high-grade IDC. There are only a few other recorded dissimilarities between IDC and ILC. ILC is mainly bigger at detection than IDC as well as is constantly estrogen and progesterone receptor positive. In addition, ILC gives metastasis to areas that are considered exceptionally rare for IDC, including the gastrointestinal system, the retroperitoneum, the gynecologic system, and the leptomeninges. In fact, metastases to the lungs, liver, and bones are considered the most familiar sites for ILC [4].

Invasive Ductal Cancer Versus Invasive Lobular Cancer

Dietzel et al. conducted a systematic comparative study on 811 patients comparing IDC and ILC over 12 years showing high diagnostic accuracy irrespective of typing [5]. They evaluated dynamic descriptors (wash-in, wash-out, continued increase, and plateau) and morphologic descriptors (shape, margin, internal structure, and internal septation). The most classic dynamic descriptor was a wash-in of the early-phase dynamic between most lesions. The most typical morphologic descriptor in most invasive breast cancers was irregular margins in ILC and IDC (sensitivity, 87.0%, and 89.9%, respectively). However, ILC was positive slightly more often than IDC for this descriptor (62.0% vs 54.5%). Overall, the morphologic profile of ILC was not significantly different from that of IDC. Benign findings included fibrocystic changes, fibroadenomas, papilloma, phyllodes tumors, and inflammations [5]. The comparison of dynamic descriptors is summarized in Table 1.

**Table 1:** Dynamic descriptors Descriptors as mentioned by Dietzel et al. [5]. PPV, positive predictive value

Invasive ductal carcinoma	Invasive lobular carcinoma
Intermediate/strong wash-in	Intermediate/strong wash-in
PPV of wash-out is 73.0%	PPV of wash-out is 40.0%

The comparison of morphological descriptors is summarized in Table 2. 

**Table 2:** Morphological descriptors Descriptors as mentioned by Dietzel et al. [5].

Invasive ductal carcinoma	Invasive lobular carcinoma
Irregular margins	Irregular margins
Easier to detect in conventional mammography or breast ultrasound due to solid nature, with less diffuse infiltration of surrounding tissue	Difficult to detect in conventional mammography or breast ultrasound
Perifocal edema is more frequent	Perifocal edema is less frequent

Clinical Importance of Preoperative MRI in Different Types of Breast Cancers and as a Screening Tool

Aydin reviewed 129 NME lesions retrospectively aiming to differentiate between the benign versus malignant lesions [6]. He found that the segmental and diffuse distribution as well as clustered-ring internal enhancement was linked to malignancy; on the other hand, the linear distribution as well as homogeneous enhancement types were associated with benignity. The plateau type of kinetic enhancement was strongly suggestive of malignancy. There was no correlation between the cystic structures and the benignity/malignancy. In fact, only lesions showing segmental distribution and diffusion restriction were associated with malignancy. Sometimes, breast cancer is undiagnosed at routine screening breast MRI studies [6,7].

Seo et al. conducted a retrospective study that evaluated undiagnosed breast cancer features on prior screening breast MRI in patients who were subsequently diagnosed with breast cancer [7]. The study showed that 33.3% of the lesions were determined to be actionable, and 66.7% were under the threshold; 85.7% of masses and non-mass enhancements were classified as actionable lesions. Mimicking physiologic enhancements (27.8%) and small lesion size (27.8%) were the most common reasons for missed cancer. MRI-detected lesions in women with BRCA1/2 mutation carriers were significantly smaller than lesions detected by a non-MRI modality. MRI could detect all lesions in BRCA1 mutation carriers [7,8]

Heywang-Köbrunner et al. conducted a meta-analysis concluding that MRI has a sensitivity of approximately 90% and a specificity of around 75% in discovering malignancy. The data showed that 90%-95% of invasive cancers enhance contrast agents versus only a part of the DCIS enhances. MRI has been the most sensitive tool for the detection of invasive cancer. They found that the exact part of DCIS detectable by MRI varies significantly, related to the chosen algorithm for MRI evaluation, on individual selection, and the detail related to histopathological correlation and verification. As a significant portion of the DCIS does not show the typical enhancement of invasive cancer, algorithms targeting a high detection rate for DCIS will have lower specificity and vice versa. The detection rate of DCIS within the thin-sliced mastectomy specimen, even small foci, was reported to be just 40% for both mammogram and MRI when compared to preoperative MRI and mammogram. MRI sensitivity for DCIS was reported to be 60%-80% in most other publications where imaging was correlated with standard histopathology [10].

MRI and mammography are both complementary in the detection of low-grade as well as high-grade DCIS. The less preferable detection of low-grade DCIS occurs with both modalities. The percentage of low-grade DCIS discovered by mammography or MRI also depends on the algorithms chosen for either modality. The study by Heywang-Köbrunner et al. revealed another systematic review comparing the accuracy of different modalities. It confirmed sensitivity of 92% and a specificity of 77.5% for the detection of malignancy. DCIS can be found incidentally during resected papilloma [10,11].

Wang et al. reviewed 175 surgically proven papilloma retrospectively. The MRI manifestations of these abnormalities were grouped into three categories: mass, NME, and occult lesion. The occult lesion was described as the presence of only ductal dilation without any enhanced masses on MRI. For a mass lesion, the mixed mass-NME lesion was considered if the linear, segmental, or regional enhanced lesion was discovered adjacent to the mass. The MRI features and clinical findings were compared by univariate and multivariate analysis between the benign papilloma and the high-risk papilloma or malignant lesions [11].

Bigila et al. evaluated preoperative MRI value for discovering breast neoplasm comparing it to traditional modalities as well as the effect of preoperative MRI on the surgical planning in patients with dense breasts, young age group, ILC, or more than one lesion. They found that enhanced breast MRI may alter the surgical treatment by either increasing the rate of mastectomy or advising for broader tissue removal. MRI can have a valuable impact on the preoperative decision if used in high-risk women. However, before surgery, it is an obligation to get the histologic type of all suspicious abnormalities discovered by MRI [12].

MRI is superior due to its ability to diagnose small lesions even before being seen on traditional imaging including mammogram and ultrasound. It plays a critical role in the workup presurgical planning as well as the staging of patients with invasive cancer including IDC and ILC. In addition, MRI is superior in discovering multifocal, multicentric, or even contralateral lesions not discovered on other modalities [13].

Contrast Agent in Enhanced Breast MRI

The pharmacokinetic profile of a contrast agent injected while performing enhanced breast MRI was evaluated by Escribano et al. in a retrospective and observational study. They evaluated 400 patients with breast cancer. Two-hundred patients underwent dynamic contrast-enhanced MRI (DCE-MRI) with the gadolinium-diethylenetriamine pentaacetic acid (Gd-DTPA) contrast (Magnevist®) and the other 200 with gadobutrol (Gadovist®) [14]. The differences between these two agents are summarized in Table 3.

**Table 3:** Differences between Gd-DTPA and gadobutrol Gd-DTPA, gadolinium-diethylenetriamine pentaacetic acid. K_trans_ is the measure of capillary permeability obtained using dynamic contrast-enhanced (DCE) MR perfusion and K_ep_ is a kinetic parameter for DCE-MRI perfusion.

Contrast agent	Gd-DTPA contrast	Gadobutrol
Relative signal intensity (enhancement)	Lower	Higher
Wash-out	58.29% (higher)	46% (lower)
Values for K_trans_ and K_ep_	Lower	Higher
Number of histologically confirmed additional malignant lesions detected	Same	Same

By reviewing the selected studies, we reviewed the importance of MRI as a high-tech tool in evaluating different types of breast cancer as shown in Table 4.

**Table 4:** Studies that focused on MRI utilization for the characterization of breast cancer HRT, hormone replacement therapy; NME, non-mass enhancement; ADC, apparent diffusion coefficient; BI-RADS, Breast Imaging Reporting and Data System

Author’s name	Year of publication	Study design	Main points
Mann [4]	2010	Prospective study	The purpose of this study was to evaluate the accuracy of preoperative MRI compared to conventional imaging in detecting breast cancer and the effect of preoperative MRI on the surgical treatment in a subgroup of women with dense breasts, young age, invasive lobular cancer or multiple lesions.
Zhang et al. [8]	2018	Comparative study	The authors compared the performance of screening mammography versus MRI in HRT users. Screening breast MRI may be a useful adjunct modality of mammography in HRT users.
Wang et al. [11]	2018	Retrospective study	The study investigated MRI features for breast papilloma and identified imaging diagnostic indicators for papilloma with high-risk or malignant lesions.
Biglia et al. [12]	2011	Retrospective study	The authors evaluated the accuracy of preoperative MRI compared to conventional imaging (including mammography and ultrasound) in detecting breast cancer as well as the effect of preoperative MRI on the surgical treatment in a subgroup of women with dense breasts, young age, invasive lobular cancer or multiple lesions.
Escribano et al. [14]	2017	Comparative study	The reason for the study is to compare the pharmacokinetic profile of gadobutrol versus Gd-DTPA in dynamic contrast-enhanced MRI in patients with breast cancer. Secondary objectives included comparing the safety profiles and diagnostic efficacy of the two contrast agents for detecting additional malignant lesions. Relative enhancement is greater with gadobutrol, but wash-out is more pronounced with Gd-DTPA. The number of additional malignant lesions detected did not differ between the two contrast agents. Both contrasts are safe.
Oh et al. [15]	2017	Retrospective study	The study described the MRI characteristics of breast cancer diagnosed during lactation and evaluate the usefulness of MRI.
Park et al. [16]	2019	Retrospective study	The study investigated the potential of diffusional kurtosis imaging and conventional diffusion-weighted imaging (DWI) in the evaluation of additional suspicious lesions at preoperative breast MRI in patients with breast cancer.
Shin et al. [17]	2016	Retrospective study	The study evaluated the diagnostic performance of fused DWI using either unenhanced (UFMR) or early postcontrast T1-weighted imaging (PCFMR) to detect and characterize breast lesions in patients with breast cancer.
Liu et al. [18]	2017	Retrospective study	This study aims to subdivide BI-RADS-MRI Category 4 lesions and to evaluate the role of Fischer’s scoring system, ADC, and Fischer’s + ADC in differential diagnosis of breast lesions.
Menezes et al. [19]	2014	Literature review	This study found that pre-operative MRI is indicated in defined groups of patients in which a potential benefit of local staging is expected, i.e., women with mammographically heterogeneous or extremely dense breasts, at high risk of breast cancer, diagnosed with invasive lobular carcinoma and/or with multifocal, multicentric or contralateral disease.
Murakami et al. [20]	2019	Literature review	The study found that MRI-detected lesions were significantly smaller than lesions detected by non-MRI modality. All lesions in BRCA1 mutation carriers could be detected by MRI.
ACR Committee [21]	2018	Educational review	This document is an educational tool designed to assist practitioners in providing appropriate radiologic care for patients. Practice parameters and technical standards are not inflexible rules or requirements of practice and are not intended, nor should they be used, to establish a legal standard of care. For these reasons and others stated in the document, the American College of Radiology and collaborating medical specialty societies caution against the use of these documents in litigation in which the clinical decisions of a practitioner are called into question.
Gonzalez-Angulo et al. [22]	2005	Retrospective study	This study concluded that young population with breast carcinoma was found to have more aggressive biologic features. Hormone receptor negativity and a family history of ovarian carcinoma were associated with worse prognosis.
DeSantis et al. [23]	2011	Educational review	In this article, the American Cancer Society provides an overview of female breast cancer statistics in the United States, including trends in incidence, mortality, survival, and screening.
Salem et al. [24]	2013	Literature review	This study showed the role of MRI in diagnosis of breast cancer in young individuals (younger than 40 years old)

**Figure 2:**
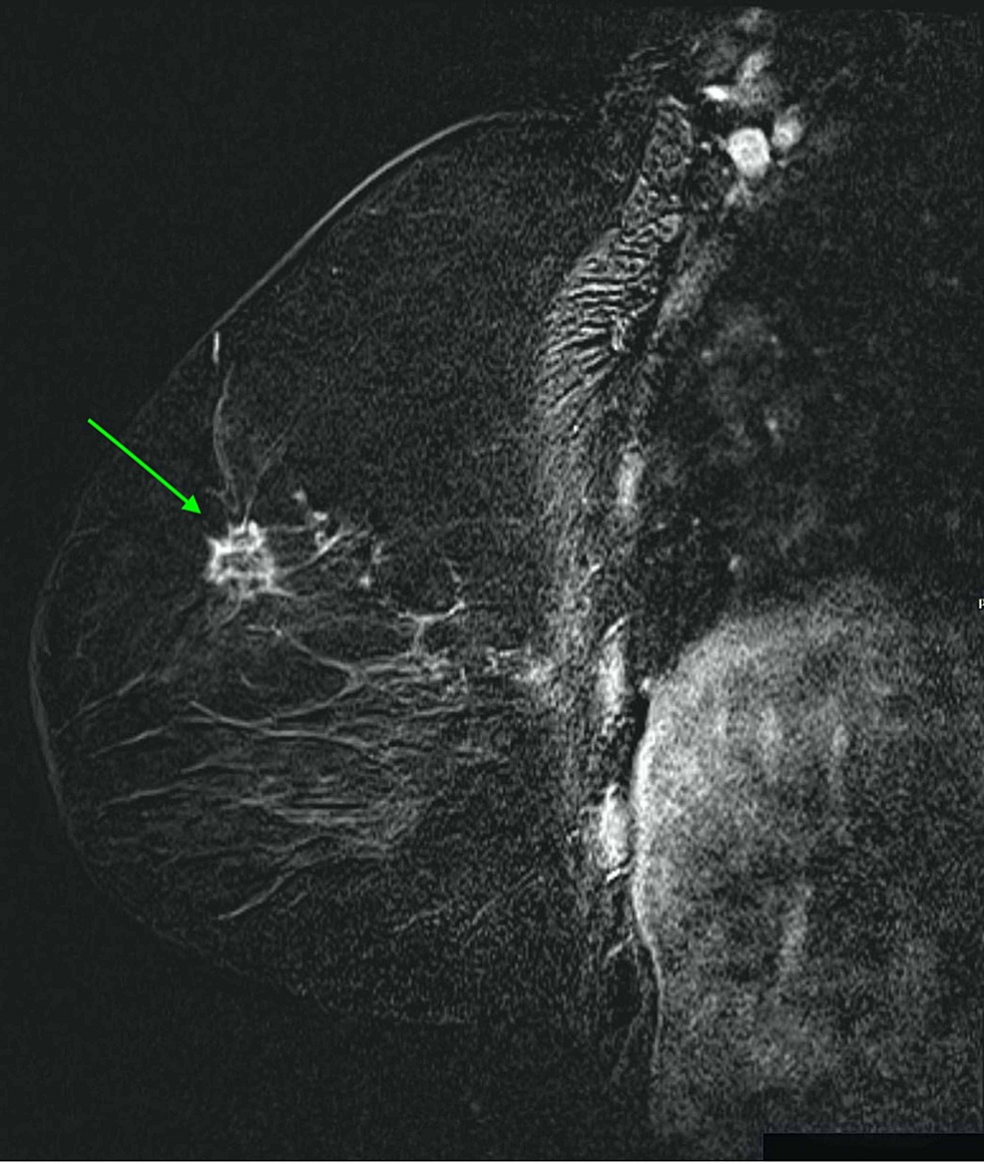
Sagittal post-contrast T1 subtraction There is an invasive ductal cancer in the upper outer quadrant of the right breast (green arrow) in an 80-year-old female appearing as an enhancing lesion with spiculated irregular margins.

**Figure 3:**
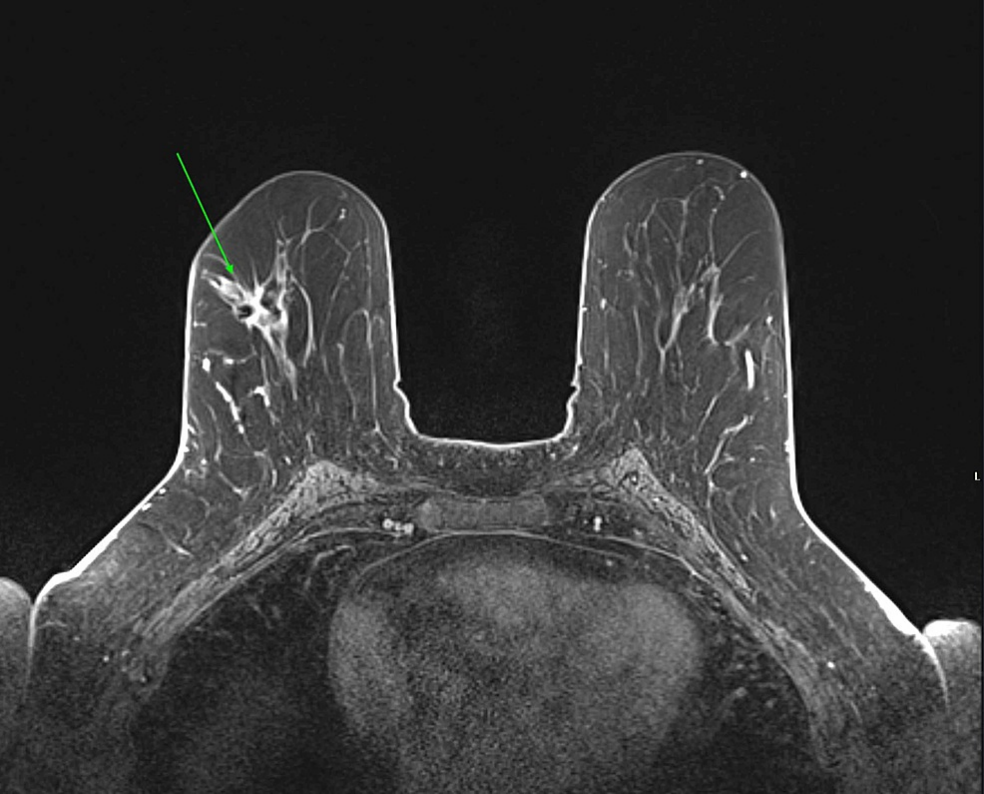
Axial post-contrast T1 subtraction There is an invasive ductal cancer in the upper outer quadrant of the right breast (green arrow) in an 80-year-old female appearing as an enhancing lesion with irregular spiculated margins.

Here is a case of an 80-year-old female with BRCA1 mutation who came to our hospital with a palpable mass in the right breast; she underwent an ultrasound-guided biopsy that came as invasive ductal carcinoma. MRI was done for better characterization of the lesion and for surgical planning as shown in Figures 2, 3.

Limitations

Our study has some limitations due to our inclusion and exclusion criteria. Since we excluded studies published in languages other than English, excluded animal studies and only included studies published in the last 10 years, this may have limited our comprehensiveness. In addition, despite doing an extensive literature search, there is also the possibility we excluded relevant articles unintentionally. Lastly, the overall sample size of our included studies was relatively small.

## Conclusions

Breast MRI is a vital imaging tool that has been increasingly used in everyday practice. Our research has confirmed that breast MRI is superior to mammogram and ultrasound in discovering early breast cancer due to its high sensitivity and specificity. However, it should not be used when it is not required. This review showed some indications for which evidence can be found in the literature.

However, we still require high-quality studies such as systematic reviews and meta-analyses to be conducted in order to determine the indications and the justification for MRI use. Future advanced technical innovations on breast MRI technique and sequences, including using diffusion weight sequence, utilizing high magnetic fields (up to 7 T), improvement of spatial resolution, and spectroscopy, are promising tools that will add to the excellence of the MRI.
